# Sudden unilateral eye pain with vision loss related to carotid stump syndrome; A case report and literature review

**DOI:** 10.1016/j.ijscr.2023.108208

**Published:** 2023-04-15

**Authors:** Saqer Alenezi, Athary Saleem, Ali Alenezi, Omar Alhajri, Salma Khuraibet, Ahmed Ameer

**Affiliations:** Vascular surgery department, Jaber Al Ahmad Hospital, Kuwait

**Keywords:** Carotid stump syndrome, Internal carotid artery occlusion, Endarterectomy, Central retinal artery occlusion, Carotid artery occlusion, Case report

## Abstract

**Introduction and importance:**

Carotid stump syndrome (CSS) is a rare cause of recurrent ipsilateral cerebrovascular accidents (CVAs) resulting from completely occluded internal carotid artery (ICA). In this condition, hemodynamic and embolic risks are related to cerebral and retinal ischemic strokes.

**Presentation of case:**

A 65-year-old gentleman with multiple comorbidities, presented to our hospital with a sudden painful unilateral vision loss of the right eye. Head CT was done upon arrival, showing no evidence of ischemic or hemorrhagic brain insult and multiple right frontoparietal old infarct lesions were detected. Central retinal artery occlusion diagnosis was confirmed by an ophthalmologist. CT angiogram of the brain and carotids was done and revealed an obliterated, thrombosed, and non-opacified right internal carotid artery from the carotid bifurcation up to intracranial petrous/foramen lacerum. After taking the patient's surgical consent, right carotid stump endarterectomy and ligation of the stump under general anesthesia was done and the postoperative period was uneventful.

**Clinical discussion:**

CSS is an uncommon underlying etiology, causing recurrent stroke events. The clinical features of this syndrome include cerebral and ophthalmology symptoms. Diagnosis of CSS relies on imaging modalities. Internal carotid artery stump surgical excision through the ipsilateral ECA endarterectomy is the gold standard for CSS treatment.

**Conclusion:**

Despite being a rare entity, CSS is a treatable cause of retinal embolic events TIAs. Therefore, it is important to raise awareness of such condition. The presented case demonstrates the diagnosis, management and prognosis of CSS.

## Highlights

• Carotid Stump Syndrome (CSS) is a rare cause of recurrent ipsilateral cerebrovascular accidents resulting from completely occluded internal carotid artery.
• The clinical features of CSS include cerebral and ophthalmology symptoms.
• In this condition, hemodynamic and embolic risks are related to cerebral and retinal ischemic strokes.
• Diagnosis of CSS relies on specific imaging modalities.
• Endarterectomy is the gold standard for CSS treatment.

## 1 Introduction

Carotid stump syndrome (CSS) is an uncommon etiology of recurrent ipsilateral cerebrovascular accidents (CVAs) resulting from completely occluded internal carotid artery (ICA) [1], [2]. CSS is detected as a proximal patent remnant located below a totally occluded internal carotid artery. In such conditions, hemodynamic and embolic risks are related to cerebral and retinal ischemic strokes, causing recurrent transient ischemic attacks (TIAs) and cerebrovascular events [2], [3], [4]. With an ipsilateral stroke rate of 3–5 % per year, the overall risk of mortality or stroke from internal carotid artery blockage is 30 % [5], [6].

Although of its rarity, this syndrome is more likely to be missed leading to improper assessment and treatment that lead to recurrent symptoms and irreversible neurological deficit [7].

Here we highlight a 65-year-old male patient, with multiple comorbidities, admitted with sudden painful vision loss of the right eye found to be caused by carotid stump syndrome. Our work has been reported in line with the SCARE 2020 criteria [8].

## 2 Case presentation

A 65-year-old gentleman, who is a known case of diabetes mellitus type 2, hypertension and ischemic heart disease, presented to our hospital with sudden painful unilateral vision loss. The patient was non-fluent aphasic with left sided facial palsy and weakness due to recurrent attacks of ischemic stroke.

His sudden onset of painful vision loss is located in the right eye, started at midnight, persistent and the patient can see only the light. There was no history of discharge, photophobia, eye swelling or redness, nausea, or vomiting. No evidence of trauma or headache was documented. He was on dual antiplatelets.

On admission, the patient was conscious, alert, and oriented. Neurological examination showed non-fluent aphasia, residual left-sided weakness, and old left facial palsy. The right-sided power was preserved while the left side was power 4 out of 5 with limb elevation unsustainability. Moreover, the sensation modalities were intact and the remaining systemic examination was unremarkable (Table 1).

**Table 1:** Patient's investigations on admission.

Patient's vital signs	Hematology		Biochemistry		Coagulation		Renal function tests	
GCS 15	WBC	13.0	ALT	46	PT	14.20	eGFR	95
BP 132/75 mmHg	Hb	130	AST	28	PT-INR	1.06	Creatinine	68
HR 67 bpm	Plt	199	ALP	129	APTT-A	47.90	Urea	3.9
RR 24 bpm	Neutrophils	12.1	GGT	67	APTT-A Ratio	1.43	Na	132
SPO2 98 % in room air	Lymphocytes	0.7	Albumin	45	–		K	4.8
Body temperature 37 °C	Monocytes	0.1	Amylase	40	–		–	
Glucose 15.5	Eosinophils	0.0	Lipase	39	–		–	
calc osmolarity 272	Basophils	0.0	Troponin-I	6	–		–	

Head CT was done upon arrival, showing no evidence of ischemic or hemorrhagic brain insult and multiple right fronto-parietal old infarct lesions were detected. At this point cardiac origin of thromboembolization was suspected, which was ruled-out by ECG and ECHO.

Central retinal artery occlusion (CRAO) diagnosis was confirmed by an ophthalmologist.

**Fig. 1:**
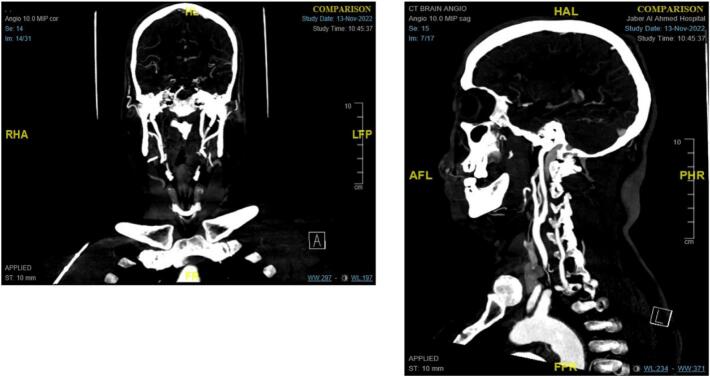
CT angiogram of brain and carotid arteries.

Then, CT angiogram of the brain and carotids was done and revealed an obliterated, thrombosed, and non-opacified right internal carotid artery from the carotid bifurcation up to intracranial petrous/foramen lacerum segments with attenuated cavernous and supra-clinoid segments (Fig. 1).

**Fig. 2:**
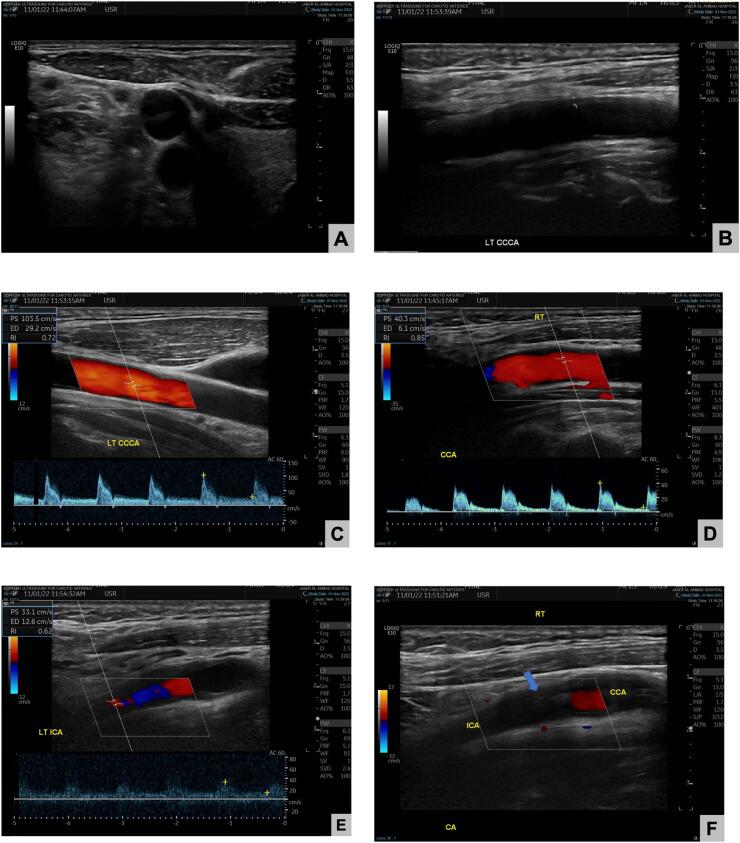
Carotid doppler scanning: A: Patent common carotid artery B: Patent left common carotid artery C: Normal left common carotid artery with normal waveforms and anterograde flow D: Retrograde flow of the right common carotid artery according to the carotid doppler. E: Average velocity along the carotid arteries and no areas of abnormal high velocity F: ICA Stump in the right ICA.

Anticoagulation started by the vascular team and further investigations were obtained such as carotid doppler for extracranial carotid system which showed (Fig. 2):

• Thickened intima/media in both sides
• Right carotid system illustrated the totally occluded right ICA by a thrombus starting from its bulb and proceeding cranially.
• Left carotid system duplex scanning revealed average velocity along the carotid arteries and no areas of abnormal high velocity.

**Fig. 3:**
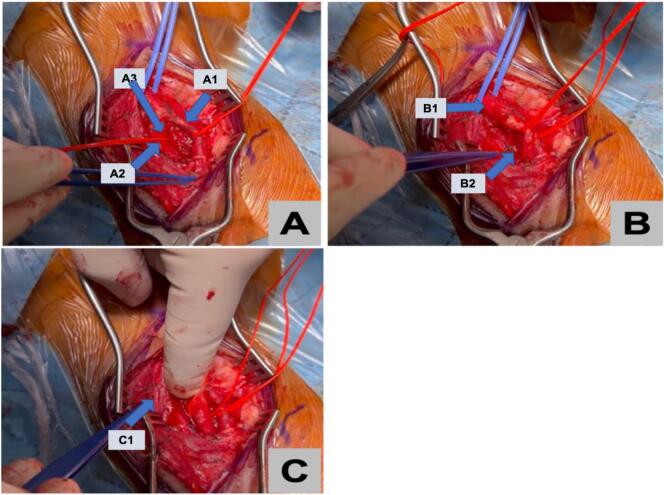
Intraoperative figures illustrating: A: A1: ECA, A2: ICA, A3: Carotid stump location B: B1: CCA, B2: vagus nerve C: C1 retracted jugular vein.

**Fig. 4:**
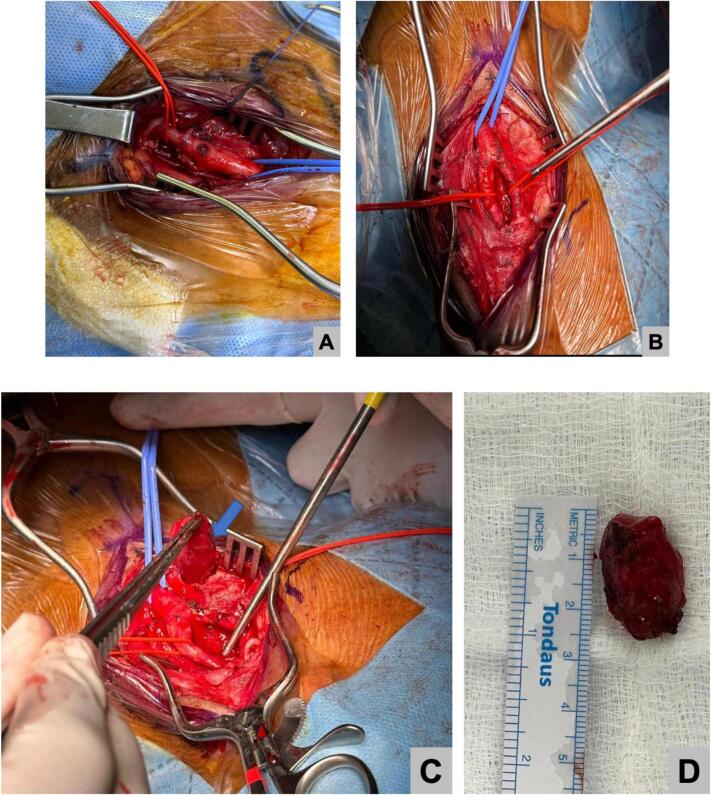
Intraoperative figures showing, A and B: clear carotid anatomy, C: Carotid stump resection D: resected specimen.

As a result, right carotid stump endarterectomy and ligation of the stump under general anesthesia was planned. During the procedure, dissection was done along the platysma and fibrous tissue bluntly, exposing the internal carotid up to the bifurcation using a self-retaining retractor. Then, the facial vein was ligated and a loop was placed superior and inferior to the common carotid. The incision made along the stump to remove the plaque, occluding the vessel. Next, blood backflow was checked and visualized within the vessel. Finally, closure of the carotid stump, muscle, subcutaneous sheath, and skin was performed. Fig. 3, Fig. 4 illustrate the intraoperative findings.

The postoperative period was uneventful and the patient discharged on postoperative day two.

## 3 Discussion

Carotid stump syndrome (CSS) is a thromboembolic event that can be considered a cryptogenic stroke. This type of stroke is mainly characterized by non-identifiable causes, accounting for up to 25 % of patients with stroke [9]. CSS is an uncommon disorder that was first described in 1978 [1], [7]. A study demonstrated that an obstructed ICA increases the risk of an ipsilateral cerebrovascular accident by 3 to 5 % per year [5], [10].

Pathophysiologically, CSS is thought to be caused by ipsilateral microembolization to the external carotid artery (ECA) and ophthalmic artery. Through retrograde collaterals, microemboli migrate to the intracranial pathways through the ECA and ophthalmic arteries [2], [7], [11]. The thromboemboli in the completely obstructed ICA can be the cause of cerebral embolization [2].

CSS is affected by both hemodynamic and/or embolic factors. In the case of hypoperfusion, generalized symptoms should be suspected by the physician. Furthermore, turbulence within the carotid stump can enhance microembolization [4], [7].

Although ipsilateral ICA obstruction without a further identifiable embolic source is the typical diagnosis of carotid stump syndrome, various other mechanisms have been identified in the presentation of this syndrome [5]. In our case, the central retinal artery occlusion can be explained by microembolization through the ophthalmic and external carotid arteries, reversing the ophthalmic artery blood flow [12].

The hemodynamic instability in the occluded ICA that causes cerebral hypoperfusion and recurrent microembolization from the thrombotic carotid stump to the brain, respectively, have been used to describe hemodynamic and embolic variables contributing to the recurrence of cerebral or retinal manifestations [3], [13].

Due to the fact that the sensitivity of the ischemic brain cells is higher to the embolic factors, microembolization persists, causing permanent cerebral clinical features, as in the current case [6], [13].

Morris-Stiff et al. documented the evaluation of carotid artery occlusion in 153 patients using ultrasound. He discovered that 10.3 % of the patients had spontaneous carotid artery recanalization and that up to 23 % of the patients suffered strokes in the ipsilateral obstructed area [10].

Diagnosis of CSS relies on specific imaging modalities such as CT angiography, MR angiography, and carotid duplex scan [3], [6], [14]. D.S.Quill et al. documented the role of the color doppler flow imaging in patient assessment, diagnosis, and the ability to develop late symptoms [14].

In the current case, a carotid duplex scan, MRA, and CTA of the brain and carotids were performed showing diagnostic findings mentioned in the case presentation section.

The definitive treatment of CSS is surgery either by open techniques or endovascular approaches [1], [3], [15]. Internal carotid artery stump surgical excision through the ipsilateral ECA endarterectomy is the gold standard for CSS treatment.

Besides optimal medical therapy, several studies recommended the surgical option for symptomatic patients [11], [15], [16]. Additionally, endovascular treatment options are effective alternatives for the recanalization and prevention of cerebral vascular accidents [3], [10].

Dulai et al. documented the effectiveness of the endovascular treatment of patients with CSS with full recovery of their neurological manifestations [1]. In 2015, a study demonstrated the role of carotid stunting in maintaining hemodynamic normality in carotid stump patients with recurrent cerebral and/or retinal clinical features [9]. Furthermore, over the past ten years, a study reported that more than 1200 carotid endarterectomies were performed, however, only two patients with stump syndrome have been surgically treated [4]. In 2016, Georgiadis et al. documented a clinical improvement in CSS patients who were treated surgically for their contralateral ICA stenosis [17].

In the reported case, right carotid stump endarterectomy and ligation of the stump were performed and the postoperative period was uneventful.

## 4 Conclusion

Although ICA occlusion is common, CSS is a rare entity. Recurrent TIAs, CVAs and retinal embolic events should raise the clinical suspicion of CSS especially in the absence of other embolic sources. The fact that CSS is treatable highlights the importance of early diagnosis. Detailed and thorough history and examination and knowledge of the timing between the symptomatology ICA occlusion are crucial to improve the outcome of the patients.

## CRediT authorship contribution statement

Saqer Alenezi: literature review, paper writing, and editing, picture editing, manuscript drafting.

Athary Saleem: paper writing, and editing, picture editing, manuscript drafting.

Ali Alenezi: paper writing and editing.

Omar Alhajri: performed surgery and picture editing.

Salma Khuraibet: paper editing, supervision, and final approval.

Ahmed Ameer: performed surgery, critical review, paper editing, supervision, and final approval.

## Funding

No funding or grant support.

## Ethical approval

Not applicable.

## Consent

Written informed consent was obtained from the patient to publish this case report and accompanying images. On request, a copy of the written consent is available for review by the Editor-in-Chief of this journal.

## Research registration

Not applicable.

## Provenance and peer review

Not commissioned, externally peer reviewed.

## Guarantor

Saqer Alenezi, M.D., General surgery department, Al-Adan Hospital, Kuwait.

## Declaration of competing interest

There is no conflict of interest including any financial or personal relationships with other people or organizations or any work influencers.
